# Supplementary material for: Spatially explicit estimation of recent migration rates in plants using genotypic data
Source: Genetics. 2025 Jan 28;229(2):iyae218. doi: 10.1093/genetics/iyae218 (PMC11796463; doi:10.1093/genetics/iyae218)
Supplement: iyae218_Supplementary_Data [file iyae218_supplementary_data.pdf]

Supplemental Material - File S1

From ***Spatially explicit estimation of recent migration rates in plants using genotypic data***,

by I.J. Chybicki & J.J. Robledo-Arnuncio.

Contents of this document:

Supplement A. Formulation of a dispersal kernel from the model parameters.

Supplement B. MCMC algorithm.

Supplement C. Monte Carlo simulations.

Supplement D. Calculation of gene migration rates from seed and pollen migration rates.

Table S1. Effect of mean population genetic differentiation ($\mu_{F_{ST}}$) and total sample size (N) on the bias and root mean square error (RMSE) of estimates of migration rates, population divergence and inbreeding, assuming microsatellite-type markers, moderate inbreeding (μ_F = 0.1) and no distance effect on migration rates.

Table S2. Effect of mean population genetic differentiation ($\mu_{F_{ST}}$) and total sample size (N) on the bias and root mean square error (RMSE) of estimates of migration rates, population divergence and inbreeding, assuming microsatellite-type markers, strong inbreeding (μ_F = 0.2) and no distance effect on migration rates.

Table S3. Effect of mean population genetic differentiation ($\mu_{F_{ST}}$) and total sample size (N) on the bias and root mean square error (RMSE) of estimates of migration rates, population divergence and inbreeding, assuming SNP-type markers, moderate inbreeding (μ_F = 0.1) and no distance effect on migration rates.

Table S4. Effect of mean population genetic differentiation ($\mu_{F_{ST}}$) and total sample size (N) on the bias and root mean square error (RMSE) of estimates of migration rates, population divergence and inbreeding, assuming SNP-type markers, strong inbreeding (μ_F = 0.2) and no distance effect on migration rates.

Table S5. Effect of the number of populations (K), total sample size (N) and marker type on the bias and root mean square error (RMSE) of estimates of migration rates, population divergence and inbreeding, assuming no inbreeding (μ_F = 0), moderate population genetic differentiation ($\mu_{F_{ST}}$ = 0.10) and no distance effect on migration rates.

Table S6. Effect of mean population genetic differentiation ($\mu_{F_{ST}}$) and magnitude of inter-population distance effects on seed and pollen migration (b_α and b_β, respectively) on the bias and root mean

square error (RMSE) of estimates of the distance effects and of seed and pollen migration rates (α_{ij} and β_{ij}, respectively), assuming a total sample size of N = 500 individuals.

Table S7. Effect of mean population genetic differentiation ($\mu_{F_{ST}}$) and magnitude of inter-population distance effects on seed and pollen migration (b_α and b_β, respectively) on the bias and root mean square error (RMSE) of estimates of the distance effects and of seed and pollen migration rates (α_{ij} and β_{ij}, respectively), assuming a total sample size of N = 250 individuals.

Table S8. Effect of the number of populations (K) and total sample size (N) on the bias and root mean square error (RMSE) of estimates of distance effects on seed and pollen migration (b_α and b_β, respectively) and of seed and pollen migration rates (α_{ij} and β_{ij}, respectively), assuming moderate population genetic differentiation ($\mu_{F_{ST}}$ = 0.10) and weak isolation by distance (b_α = b_β = 1.2062).

Table S9. Posterior estimates of recent seed (α_{jk}) and pollen (β_{jk}) migration rates among nine *Taxus baccata* remnant populations on the Low Beskids (Poland).

Figure S1. Posterior estimates of pre-migration divergence rates (F_{ST}^{j}) for nine remnant populations of *Taxus baccata*.

Figure S2. Posterior estimates of allelic drop-out rates for the 20 microsatellite loci used in the *Taxus baccata* case study.

Supplement A. Formulation of a dispersal kernel from the model parameters.

Given the λ, τ and b (seed or pollen) migration parameters (see Equations 6 and 7 in the main text), it is possible to obtain a probability density function of dispersal distance (x) from the source, i.e. a dispersal kernel. In general, the expression $\exp\left(-b \log\left(1 + d_{ij}\right)\right)$ used in Equation 7 corresponds to an improper (infinite integral) power-law kernel of the form $f(x) = (1 + x)^{-b}$. It is possible, however, to obtain a proper density function by assuming a threshold distance *t* beyond which the probability of migration becomes virtually null and using then the normalizing constant

$$C(t) = \begin{cases} \dfrac{1}{2\pi(t - \log(1 + t))} & \text{if } b = 1 \\ \dfrac{t + 1}{2\pi\left((1 + t)\log(1 + t) - t\right)} & \text{if } b = 2 \\ \dfrac{(b - 2)(b - 1)}{2\pi(1 + t)^b\left((1 + t)^b - (1 + t)(bt - t + 1)\right)} & \text{otherwise} \end{cases}$$

The resulting dispersal kernel, involving both local and migrant propagules, takes the form:

$$f(x; \lambda, \tau, b, t) = (\lambda + (1 - \lambda)\tau)\mathbb{I}_0(x) + (1 - \lambda)(1 - \tau)\frac{C(t)}{(1 + x)^b}$$

where $\mathbb{I}_0(x)$ is an indicator function that equals 1 if $x = 0$, and 0 otherwise.

The cumulative (isotropic) density function is in turn given by:

$$F(x; \lambda, \tau, b, t) = \lambda + (1 - \lambda)\tau + (1 - \tau)(1 - \tau)F_m(x; b, t)$$

where

$$F_m(x; b, t) = \begin{cases} \dfrac{\log(1 + x) - x}{\log(1 + t) - t} & \text{if } b = 1 \\ \dfrac{(1 + t)\left((1 + x)\log(1 + x) - r\right)}{(1 + x)\left((1 + t)\log(1 + t) - t\right)} & \text{if } b = 2 \\ \dfrac{\left((1 + x)^b - (1 + x)(bx - x + 1)\right)\left(\dfrac{1 + x}{1 + t}\right)^b}{(1 + t)^b - (1 + t)(bt - t + 1)} & \text{otherwise} \end{cases}$$

And the mean migration distance can be calculated as

$$\delta = (1 - \lambda)(1 - \tau)\delta_m$$

where

$$\delta_m = \begin{cases} \dfrac{2\log(1+t) - t(2+t)}{2(t - \log(1+t))} & \text{if } b = 1 \\ \dfrac{2(1+t)\log(1+t) - t(2+t)}{t - (1+t)\log(1+t)} & \text{if } b = 2 \\ \dfrac{2(1+t)^2\log(1+t) - t(3t+2)}{t^2} & \text{if } b = 3 \\ \dfrac{2(1+t)^b - (1+t)\big(b^2t^2 + bt(2-3t) + 2(t^2 - t + 1)\big)}{(b-3)\big((1+t)^b - (1+t)(bt - t + 1)\big)} & \text{otherwise} \end{cases}$$

Supplement B. MCMC algorithm.

Data dimensions

Here, for the sake of clarity, we define all data dimensions that enter to particular Gibbs sampler steps. There are N individuals sampled from K populations, genotyped at L marker loci. The l-th marker has A_l alleles. The number of loci with missing data in the i-th individual's genotype is M_i. And the number of heterozygous loci at the i-th individual's genotype is H_i.

Auxiliary variables

To simplify the estimation, we introduced a set of auxiliary variables that atomize the model structure into easily tractable pieces of information. Specifically, let $\mathbf{\Psi} = \{\mathbf{o}, \mathbf{r}, \mathbf{x}, \mathbf{w}\}$ be the vector of unobserved auxiliary variables. $\mathbf{o}$ is a vector of indicators pointing at individual population origins $\mathbf{o} = \{\mathbf{o}_1, \mathbf{o}_2, \dots, \mathbf{o}_N\}$, where $\mathbf{o}_i = \{o_{i1}, o_{i2}\}$, with o_{i1} and o_{i2} indicating the population origin of the i-th individual's maternal and paternal gametes, respectively. $\mathbf{r} = \{\mathbf{r}_1, \mathbf{r}_2, \dots, \mathbf{r}_N\}$ is a vector of indicators pointing at the population origin of alleles within individual genotypes, with $\mathbf{r}_i = \{r_{i1}, r_{i2}, \dots, r_{iL}\}$ and $r_{il} = \{r_{il1}, r_{il2}\}$, where r_{il1} and r_{il2} indicate the population origin of the first and second alleles at the l-th locus of the i-th individual. $\mathbf{x} = \{\mathbf{x}_1, \mathbf{x}_2, \dots, \mathbf{x}_N\}$, where $\mathbf{x}_i = \{x_{i1}, x_{i2}, \dots, x_{iL}\}$, with x_{il} being a binary indicator equal to 1 if alleles at the l-th locus of the i-th individual's genotype are identical by descent (IBD; due to inbreeding), and 0 otherwise. Finally, $\mathbf{w} = \{\mathbf{w}_1, \mathbf{w}_2, \dots, \mathbf{w}_N\}$, where $\mathbf{w}_i = \{w_{i1}, w_{i2}, \dots, w_{iL}\}$ and w_{il} is a binary indicator equal to 1 if

homozygosity at the l-th locus of the i-th individual's genotype is due to allelic drop-out, and 0 otherwise.

MCMC algorithm steps

In the Gibbs sampler (Casella & George, 1992), a set of conditional probability distributions is used to update sequentially all elements of the model, including model parameters and auxiliary variables. We used an algorithm that is broadly similar to those used in other methods dealing with "genetic" mixture models (e.g. Pritchard et al. 2000, Vogl et al. 2002, Chybicki et al. 2011). Given a satisfactory number of cycles, an approximation to the full posterior distribution is obtained from a sequence of random updates of model parameters. A single cycle of the sampler begins by drawing $\mathbf{\Psi}$ based on current parameter values. Subsequently, values of model parameters are drawn given the updated $\mathbf{\Psi}$. In the next step, hyper-parameters are updated, given updated model parameters. Finally and optionally, migration models (spatial vs. non-spatial) used as priors for migration rates are updated using the Reversible Jump Markov Chain Monte Carlo approach. These steps are now described in more detail.

Step 1. Updating auxiliary variables

1.1. Updating individual origins

For the i-th individual, the origin vector $o_i = \{j,k\}$ is sampled from a categorical distribution where $o_i = \{j,k\}$ has the probability

$$\mathrm{Pr}\left(o_i = \{j,k\} | G_i, \boldsymbol{\alpha}_{s_i}, \boldsymbol{\beta}, \mathbf{p}, F_i, \boldsymbol{\varepsilon}\right) = \frac{\alpha_{s_i j}\beta_{jk}\,\mathrm{Pr}_{jk}(G_i\,|\mathbf{p}, F_i, \boldsymbol{\varepsilon})}{\sum_{j'=1}^{K}\sum_{k'=1}^{K}\alpha_{s_i j'}\beta_{j'k'}\,\mathrm{Pr}_{j'k'}(G_i\,|\mathbf{p}, F_i, \boldsymbol{\varepsilon})}$$

1.2. Updating individual indicator variables

For the i-th individual, with the origin $o_i = \{j,k\}$, vector elements $\mathbf{r}_i$, $\mathbf{x}_i$, and $\mathbf{w}_i$ are sampled simultaneously (in a block), locus by locus. Let $G_{il} = \{g_{il1}, g_{il2}\}$ be the i-th individual's genotype at the l-th locus. For the l-th locus, four sampling sub-algorithms can be distinguished depending on o_i and G_{il}:

i) if $g_{il1} = g_{il2}$ and $j = k$:

Given the origin o_i, the total probability for G_{il} is $\Pr(G_{il}) = F_i p_{jlg_{il1}} + (1 - F_i)\left(p_{jlg_{il1}}^2 + \varepsilon_l p_{jlg_{il1}}(1 - p_{jlg_{il1}})\right)$. Homozygosity is due to inbreeding (identity-by-descent identity, $x_{il} = 1$) with probability $F_i p_{jlg_{il1}} / \Pr(G_{il})$. In that case, a single allele copy is counted in the gene pool of population j (conventionally denoted with $r_{il} = \{j, 0\}$), and there is no indication of allelic dropout ($w_{il} = 0$). Alternatively, homozygosity is a result of random sampling of two allele copies ($x_{il} = 0$) with probability $(1 - F_i)p_{jlg_{il1}}^2 / \Pr(G_{il})$. In that case, two allele copies are counted in the gene pool of population j ($r_{il} = \{j, j\}$), and there is no indication of allelic dropout ($w_{il} = 0$). Finally, homozygosity is due to allelic dropout with probability $(1 - F_i)\varepsilon_l p_{jlg_{il1}}(1 - p_{jlg_{il1}}) / \Pr(G_{il})$. In that case $w_{il} = 1$, there is no indication of inbreeding ($x_{il} = 0$), and one allele copy is counted in the gene pool of population j ($r_{il} = \{j, 0\}$) (since the other allele is treated as missing data).

A block of (r_{il}, x_{il}, w_{il}) is sampled randomly from the categorical distribution

r_{il}	x_{il}	w_{il}	$\Pr(r_{il}, x_{il}, w_{il})$
$\{j, 0\}$	1	0	$F_i p_{jlg_{il1}} / \Pr(G_{il})$
$\{j, j\}$	0	0	$(1 - F_i)p_{jlg_{il1}}^2 / \Pr(G_{il})$
$\{j, 0\}$	0	1	$(1 - F_i)\varepsilon_l p_{jlg_{il1}}(1 - p_{jlg_{il1}}) / \Pr(G_{il})$

ii) if $g_{il1} \neq g_{il2}$ and $j = k$:

The total probability for G_{il} is $\Pr(G_{il}) = (1 - F_i)(1 - \varepsilon_l)2p_{jlg_{il1}}p_{jlg_{il2}}$. In this case, however, as there is no indication of inbreeding or allelic dropout, $x_{il} = 0$ and $w_{il} = 0$, and both (visible) alleles are counted in population j ($r_{il} = \{j, j\}$).

iii) if $g_{il1} = g_{il2}$ and $j \neq k$:

Here, under the assumption that the identity by descent occurs only if alleles are drawn from the same population, $x_{il} = 0$ and the total probability for G_{il} is $\Pr(G_{il}) = p_{jlg_{il1}}p_{klg_{il1}} +$

$\frac{\varepsilon_l}{2}\left(p_{jlg_{il1}}\left(1-p_{klg_{il1}}\right)+p_{klg_{il1}}\left(1-p_{jlg_{il1}}\right)\right)$. Consequently, homozygosity is an effect of random draw of identical alleles from two different populations with probability $p_{jlg_{il1}}p_{klg_{il1}}/\Pr(G_{il})$. In that case $r_{il} = \{j,k\}$ and $w_{il} = 0$. Alternatively, homozygosity is due to allelic dropout and the visible allele g_{il1} is drawn from population j with probability $\frac{\varepsilon_l}{2}p_{jlg_{il1}}\left(1-p_{klg_{il1}}\right)/\Pr(G_{il})$. In that case $r_{il} = \{j,0\}$ and $w_{il} = 1$. Finally, homozygosity is due to allelic dropout and the visible allele g_{il1} is drawn from population k with probability $\frac{\varepsilon_l}{2}p_{klg_{il1}}\left(1-p_{jlg_{il1}}\right)/\Pr(G_{il})$. In that case $r_{il} = \{k,0\}$ and $w_{il} = 1$.

A block of (r_{il}, w_{il}) is sampled randomly from the categorical distribution

r_{il}	w_{il}	$\Pr(r_{il}, x_{il}, w_{il})$
$\{j,k\}$	0	$p_{jlg_{il1}}p_{klg_{il1}}/\Pr(G_{il})$
$\{j,0\}$	1	$\frac{\varepsilon_l}{2}p_{jlg_{il1}}\left(1-p_{klg_{il1}}\right)/\Pr(G_{il})$
$\{k,0\}$	1	$\frac{\varepsilon_l}{2}p_{klg_{il1}}\left(1-p_{jlg_{il1}}\right)/\Pr(G_{il})$

iv) if $g_{il1} \neq g_{il2}$ and $j \neq k$:

In this last case, we again have $x_{il} = 0$, since $j \neq k$, and also $w_{il} = 0$, since $g_{il1} \neq g_{il2}$. The total probability for G_{il} is $\Pr(G_{il}) = (1-\varepsilon_l)\left(p_{jlg_{il1}}p_{klg_{il2}} + p_{klg_{il1}}p_{jlg_{il2}}\right)$. Here, only the allele origin vector r_{il} is inferred, taking into account two possible scenarios. Consequently, the vector r_{il} is sampled randomly from the categorical distribution

r_{il}	$\Pr(r_{il}, x_{il}, w_{il})$
$\{j,k\}$	$(1-\varepsilon_l)p_{jlg_{il1}}p_{klg_{il2}}/\Pr(G_{il})$
$\{k,j\}$	$(1-\varepsilon_l)p_{klg_{il1}}p_{jlg_{il2}}/\Pr(G_{il})$

Step 2. Updating model parameters

2.1. Updating seed and pollen migration rates

Having the matrix of individual origins $\mathbf{o}$, the seed migration vector $\boldsymbol{\alpha}_j$ for the j-th population is sampled directly from the Dirichlet distribution

$$\boldsymbol{\alpha}_j \sim Dirichlet\left(\mathbf{n}_j + \boldsymbol{\pi}_j^{(\alpha)} \times \frac{(1-\gamma_\alpha)}{\gamma_\alpha}\right),$$

where $\boldsymbol{\pi}_j^{(\alpha)} = \{\pi_{j1}^{(\alpha)}, \pi_{j2}^{(\alpha)}, \dots, \pi_{jK}^{(\alpha)}\}$ is a vector of expected seed migration rates for the j-th population, γ_α is a dispersion hyper-parameter, and $\mathbf{n}_j = \{n_{j1}, n_{j2}, \dots, n_{jK}\}$ has elements $n_{jk} = \sum_{i=1}^{N} \delta_{js_i}\delta_{ko_{i1}}$, where δ_{ij} is the Kronecker delta (i.e. δ_{js_i} equals 1 if the i-th individual's sampling location $s_i = j$, and 0 otherwise). Consequently, n_{jk} is the number of individuals with the sampling location j and seed origin k.

Analogously, for the j-th population, the pollen migration vector $\boldsymbol{\beta}_j$ is sampled directly from the Dirichlet distribution

$$\boldsymbol{\beta}_j \sim Dirichlet\left(\mathbf{m}_j + \boldsymbol{\pi}_j^{(\beta)} \times \frac{(1-\gamma_\beta)}{\gamma_\beta}\right),$$

where $\boldsymbol{\pi}_j^{(\beta)} = \{\pi_{j1}^{(\beta)}, \pi_{j2}^{(\beta)}, \dots, \pi_{jK}^{(\beta)}\}$ is a vector of expected pollen migration rates for the j-th population, γ_β is a dispersion hyper-parameter, and $\mathbf{m}_j = \{m_{j1}, m_{j2}, \dots, m_{jK}\}$ with the k-th element $m_{jk} = \sum_{i=1}^{N} \delta_{jo_{i1}}\delta_{ko_{i2}}$. Here, m_{jk} is the number of individuals with seed origin j and pollen origin k.

2.2. Updating individual inbreeding

Having the indicator vector $\mathbf{x}_i$, the inbreeding coefficient for the i-th individual is sampled from the beta distribution

$$F_i \sim Beta(a_i, b_i)$$

where $a_i = \sum_{l=1}^{L} x_{il} + \frac{\mu_F(1-\gamma_F)}{\gamma_F}$, $b_i = L - M_i + \frac{(1-\mu_F)(1-\gamma_F)}{\gamma_F}$ and μ_F and γ_F are the grand mean and dispersion hyper-parameters, respectively. Here, $\sum_{l=1}^{L} x_{il}$ is the number of loci with IBD alleles, and $L - M_i$ is the number of loci with non-IBD alleles (accounting for missing data).

2.3. Updating allele frequencies within populations

Allele frequencies at the l-th locus within the j-th population are sampled directly from the Dirichlet distribution

$$\mathbf{p}_{jl} \sim Dirichlet\left(\mathbf{z}_{jl} + \mathbf{q}_l \times \frac{(1 - F_{STj})}{F_{STj}}\right)$$

where $\mathbf{q}_l$ are the global allele frequencies at locus l, F_{STj} is the genetic divergence of the j-th population, and $\mathbf{z}_{jl} = \{z_{jl1}, z_{jl2}, \dots, z_{jlA_l}\}$, with $z_{jla} = \sum_{i=1}^{N}(\delta_{jr_{il1}}\delta_{ag_{il1}} + \delta_{jr_{il2}}\delta_{ag_{il2}})$. The latter expression in parentheses summarizes the information about single alleles' origins ($\mathbf{r}$). For example, the product $\delta_{jr_{il1}}\delta_{ag_{il1}}$ equals 1 if the first allele at the l-th locus in the i-th individual's genotype is a and has the origin in the j-th population, and 0 otherwise. Consequently, z_{jla} is the total number of copies, computed across all individuals, of the a-th allele having the origin in the j-th population.

2.4. Updating allelic dropout rates

Having the $\mathbf{w}$ matrix, the allelic drop-out rate for the l-th locus is drawn from the beta distribution

$$\varepsilon_l \sim Beta(a_l, b_l)$$

where $a_l = \sum_{i=1}^{N} w_{il} + 0.01$ and $b_l = H_i + 0.01$.

Step 3. Updating hyper-parameters

The hyper-parameters are updated using the Metropolis algorithm (Metropolis 1953). In this updating scheme, for a given hyper-parameter ψ, a new value (ψ^*) is proposed from a symmetric proposal distribution (e.g. a normal distribution centered at the current hyper-parameter value, i.e. ψ^c). Next, ψ^* is accepted as an updated parameter value with (acceptance) probability $A = \min\left(1, \frac{P(\boldsymbol{\varphi}|\psi^*)P(\psi^*)}{P(\boldsymbol{\varphi}|\psi^C)P(\psi^c)}\right)$, where $P(\boldsymbol{\varphi}|\psi^*)$ is the probability of observing a family of parameters $\boldsymbol{\varphi} = \{\varphi_1, \varphi_2, \dots\}$ (e.g. a vector of individual inbreeding coefficients) given the hyper-parameter ψ^* value and $P(\psi^*)$ is the prior probability assigned to ψ^*. To compute $P(\boldsymbol{\varphi}|\psi^*)$, we assumed that individual parameter values represent independent draws from the same distribution. Consequently,

$P(\boldsymbol{\varphi}|\psi^*) = \prod_i P(\varphi_i|\psi^*)$. For hyper-parameters constrained within the (0, 1) interval, new values were proposed from the uniform distribution centered at the current parameter value and the range from $\psi^c - r_\psi$ to $\psi^c + r_\psi$. For real-valued hyper-parameters (regression slopes in the spatial migration model), new values were proposed from the normal distribution with mean ψ^c and variance σ_ψ^2. The proposal parameters r_ψ and σ_ψ^2 are adjusted to get the mean acceptance rate between 25 and 45% (see "Pilot-tuning the proposals" for details). In the case of the uniform proposal distribution, we use sampling with reflection (i.e. $\psi^* = -\psi^*$ if $\psi^* < 0$ and $\psi^* = 2 - \psi^*$ if $\psi^* > 1$) to satisfy $\psi \in (0,1)$.

3.1. Updating genetic divergence rates

For the j-th population, F_{STj}^*, drawn from the uniform distribution with proposal parameter $r_{F_{ST}}$, is accepted with probability

$$A = \min\left(1, \frac{P\left(\boldsymbol{\varphi}\middle|F_{STj}^*\right)P\left(F_{STj}^*\right)}{P\left(\boldsymbol{\varphi}\middle|F_{STj}^c\right)P\left(F_{STj}^c\right)}\right)$$

where $P\left(\boldsymbol{\varphi}\middle|F_{STj}^*\right) = \prod_{l=1}^{L} Dirichlet(\,\mathbf{p}_{jl}; \mathbf{a}_{jl})$ and $P\left(F_{STj}^*\right) = Gamma_{0,1}\left(F_{STj}^*; \mu_{F_{ST}}, 1\right)$. The parameter vector of the Dirichlet distribution is $\mathbf{a}_{jl} = \mathbf{q}_l(1 - F_{STj})/F_{STj}$. Here, $Gamma_{0,1}$ denotes a gamma distribution truncated at 1.

3.2. Updating ancestral (global) allele frequencies

For the l-th locus, the vector of ancestral alleles $\mathbf{q}_l$ is updated using a block sampling to satisfy $\sum_{a=1}^{A_l} q_{la} = 1$ and $\forall q_{la} \in (0,1)$. To propose $\mathbf{q}_l^*$, two allele indicators x and y are chosen at random such that $x \neq y$ and $x, y \in (1, A_l)$. The value of q_{lx}^* is drawn from the uniform distribution with the proposal parameter r_{q_l}. The value $q_{ly}^* = q_{ly}^c - (q_{lx}^* - q_{lx}^c)$. If $q_{ly}^* < 0$ or $q_{ly}^* > 1$, the proposed vector $\mathbf{q}_l^*$ is automatically rejected. Otherwise, $\mathbf{q}_l^*$ is accepted with probability

$$A = \min\left(1, \frac{P(\boldsymbol{\varphi}|\mathbf{q}_l^*)P(\mathbf{q}_l^*)}{P(\boldsymbol{\varphi}|\mathbf{q}_l^c)P(\mathbf{q}_l^c)}\right)$$

where $P(\boldsymbol{\varphi}|\mathbf{q}_l) = \prod_{j=1}^{K} Dirichlet(\mathbf{p}_{jl}; \mathbf{a}_{jl})$ and $P(\mathbf{q}_l^*) = Dirichlet(\mathbf{1})$. The parameter vector of the Dirichlet distribution is $\mathbf{a}_{jl} = \mathbf{q}_l(1 - F_{STj})/F_{STj}$.

3.3. Updating isolation parameters

Isolation parameters for seed and pollen migration are sampled independently. A new value of τ_α^* is drawn from the uniform distribution with proposal parameter r_{τ_α}. Then, τ_α^* is accepted with probability

$$A = \min\left(1, \frac{P(\boldsymbol{\varphi}|\tau_\alpha^*)P(\tau_\alpha^*)}{P(\boldsymbol{\varphi}|\tau_\alpha^c)P(\tau_\alpha^c)}\right)$$

where $P(\boldsymbol{\varphi}|\tau_\alpha^*) = \prod_{j=1}^{K} Dirichlet(\boldsymbol{\alpha}_j; \boldsymbol{\pi}_{\alpha j}^*, \gamma_\alpha)$ and $P(\tau_\alpha^*) = Beta(\mathbf{1})$. Here, $\boldsymbol{\pi}_{\alpha j}$ is a vector of expected seed migration rates into population j, with every single element $\pi_{\alpha jk}$ being a function of τ_α (see equation 6 in the main text).

Analogously, a new value of τ_β^* is drawn from the uniform distribution with proposal parameter r_{τ_β}. Then, τ_β^* is accepted with probability

$$A = \min\left(1, \frac{P(\boldsymbol{\varphi}|\tau_\beta^*)P(\tau_\beta^*)}{P(\boldsymbol{\varphi}|\tau_\beta^c)P(\tau_\beta^c)}\right)$$

where $P(\boldsymbol{\varphi}|\tau_\beta^*) = \prod_{j=1}^{K} Dirichlet(\boldsymbol{\beta}_j; \boldsymbol{\pi}_{\beta j}^*, \gamma_\beta)$ and $P(\tau_\beta^*) = Beta(\mathbf{1})$. Here, $\boldsymbol{\pi}_{\beta j}$ is a vector of expected pollen migration rates into population j, with every single element $\pi_{\beta jk}$ being a function of τ_β.

3.4. Updating distance effects (optional)

Distance effect parameters (regression slopes) for seed and pollen migration are sampled independently. A new value of b_α^* is drawn from a normal distribution with mean b_α^c and variance $\sigma_{b_\alpha}^2$. Then, b_α^* is accepted with probability

$$A = \min\left(1, \frac{P(\boldsymbol{\varphi}|b_\alpha^*)P(b_\alpha^*)}{P(\boldsymbol{\varphi}|b_\alpha^c)P(b_\alpha^c)}\right)$$

where $P(\boldsymbol{\varphi}|b_\alpha^*) = \prod_{j=1}^{K} Dirichlet(\boldsymbol{\alpha}_j; \boldsymbol{\pi}_{\alpha j}^*, \gamma_\alpha)$ and $P(b_\alpha^*) = Normal(0{,}100)$. Note that the expected seed migration rate vector $\boldsymbol{\pi}_{\alpha j}^*$ is a function of b_α.

The parameter b_β is updated analogously. A new value of b_β^* is drawn from a normal distribution with mean b_β^c and variance $\sigma_{b_\beta}^2$. Then, b_β^* is accepted with probability

$$A = \min\left(1, \frac{P(\boldsymbol{\varphi}|b_\beta^*)P(b_\beta^*)}{P(\boldsymbol{\varphi}|b_\beta^c)P(b_\beta^c)}\right)$$

where $P(\boldsymbol{\varphi}|b_\beta^*) = \prod_{j=1}^{K} Dirichlet(\boldsymbol{\alpha}_j; \boldsymbol{\pi}_{\beta j}^*, \gamma_\beta)$ and $P(b_\beta^*) = Normal(0{,}100)$.

In addition, to avoid under- or overflow errors, b^* was automatically rejected if $abs(b^*) > 20$. If geographic coordinates for populations are not provided, b_α and b_β are automatically fixed at 0. Similarly, b_α and/or b_β are fixed at 0 if the user does not choose the spatial migration model as a prior for seed and/or pollen migration.

3.5. Updating migration dispersion parameters

A new value of γ_α^* is drawn from the uniform distribution with proposal parameter r_{γ_α}. Then, γ_α^* is accepted with probability

$$A = \min\left(1, \frac{P(\boldsymbol{\varphi}|\gamma_\alpha^*)P(\gamma_\alpha^*)}{P(\boldsymbol{\varphi}|\gamma_\alpha^c)P(\gamma_\alpha^c)}\right)$$

where $P(\boldsymbol{\varphi}|\gamma_\alpha^*) = \prod_{j=1}^{K} Dirichlet(\boldsymbol{\alpha}_j; \boldsymbol{\pi}_{\alpha j}, \gamma_\alpha^*)$ and $P(\gamma_\alpha^*) = Beta(\mathbf{1})$. Here, $\boldsymbol{\pi}_{\alpha j}$ is the vector of expected seed migration rates into population j (given by equation 6 in the main text).

Analogously, a new value of γ_β^* is drawn from the uniform distribution with proposal parameter r_{γ_β}. Then, γ_β^* is accepted with probability

$$A = \min\left(1, \frac{P(\boldsymbol{\varphi}|\gamma_\beta^*)P(\gamma_\beta^*)}{P(\boldsymbol{\varphi}|\gamma_\beta^c)P(\gamma_\beta^c)}\right)$$

where $P(\boldsymbol{\varphi}|\gamma_\beta^*) = \prod_{j=1}^{K} Dirichlet(\boldsymbol{\beta}_j; \boldsymbol{\pi}_{\beta j}, \gamma_\beta^*)$ and $P(\gamma_\beta^*) = Beta(\mathbf{1})$.

3.6. Updating inbreeding mean and dispersion

Hyper-parameters μ_F and γ_F defining the beta prior distribution assigned to inbreeding are updated independently. Such a sampling scheme was empirically proved to generate a correct approximated posterior distribution (e.g. Chybicki et al. 2011). First, a new value of μ_F^* was drawn from the uniform distribution with proposal parameter r_{μ_F}. Then, μ_F^* was accepted with probability

$$A = \min\left(1, \frac{P(\boldsymbol{\varphi}|\mu_F^*)P(\mu_F^*)}{P(\boldsymbol{\varphi}|\mu_F^c)P(\mu_F^c)}\right)$$

where $P(\boldsymbol{\varphi}|\mu_F^*) = \prod_{i=1}^{N} Beta(F_i; \mu_F^*, \gamma_F)$ and $P(\mu_F^*) = Beta(\mathbf{1})$.

Similarly, a new value of γ_F^* was drawn from the uniform distribution with proposal parameter r_{γ_F}. Then, γ_F^* was accepted with probability

$$A = \min\left(1, \frac{P(\boldsymbol{\varphi}|\gamma_F^*)P(\gamma_F^*)}{P(\boldsymbol{\varphi}|\gamma_F^c)P(\gamma_F^c)}\right)$$

where $P(\boldsymbol{\varphi}|\gamma_F^*) = \prod_{i=1}^{N} Beta(F_i; \mu_F, \gamma_F^*)$ and $P(\gamma_F^*) = Beta(\mathbf{1})$.

3.7. Updating mean population divergence

A new value of $\mu_{F_{ST}}^*$ was drawn from the uniform distribution with proposal parameter $r_{\mu_{F_{ST}}}$, and it is subsequently accepted with probability

$$A = \min\left(1, \frac{P(\boldsymbol{\varphi}|\mu_{F_{ST}}^*)P(\mu_{F_{ST}}^*)}{P(\boldsymbol{\varphi}|\mu_{F_{ST}}^c)P(\mu_{F_{ST}}^c)}\right)$$

where $P(\boldsymbol{\varphi}|\mu_{F_{ST}}^*) = \prod_{j=1}^{K} Gamma_{0,1}(F_{ST\,j}; \mu_{F_{ST}}^*, 1)$ and $P(\mu_{F_{ST}}^*) = Beta(\mathbf{1})$.

Step 4. Updating migration models (optional)

This step involves jumps between the spatial and non-spatial migration model. Therefore, it is only included in the estimation procedure if spatial coordinates are provided. The spatial model for seed and pollen migration refers to the case when $b_\alpha \neq 0$ and $b_\beta \neq 0$, respectively. Otherwise, the migration model is non-spatial.

Jumps between migration models represent jumps between $M_0 \equiv (b = 0)$ and $M_1 \equiv (b \neq 0)$, and are analogous to jumps between regression models with a single explanatory variable. Therefore, our implementation follows that in Foll & Gaggiotti (2006). Let's assume that the current seed migration model is $M_\alpha^c = M_0$. In the current cycle, the proposed alternative model is then $M_\alpha^* = M_1$, equivalent to drawing a random value b_α from a specified distribution $Q(b_\alpha)$, which is accepted with probability

$$A = \min\left(1, \frac{P(\boldsymbol{\varphi}|b_\alpha \neq 0)P(b_\alpha)P(M_1)}{P(\boldsymbol{\varphi}|b_\alpha = 0)Q(b_\alpha = 0)P(M_0)}\right)$$

where $P(\boldsymbol{\varphi}|b_\alpha) = \prod_{j=1}^{K} Dirichlet(\boldsymbol{\alpha}_j; \boldsymbol{\pi}_{\alpha j}, \gamma_\alpha)$ and $P(b_\alpha) = Normal(0{,}100)$. $P(M_m)$ is the prior probability of the model M_m; here, we assumed that the two model are a priori equally likely, yielding $P(M_0) = P(M_1) = \frac{1}{2}$. For the proposal distribution $Q(b_\alpha)$, we chose a normal distribution with the mean and variance estimated as the empirical mean and variance of b_α in the pilot run based on the saturated model (M_1) (see Foll & Gaggiotti 2006). The reverse jump (from M_1 to M_0) is realized by setting $b_\alpha = 0$, and accepting the proposed model the probability $min(1{,}1/A)$. Jumps between pollen migration models are performed analogously.

Pilot-tuning the proposals

During the burn-in period (by default first 10,000 cycles), every 1,000 cycles the parameters of proposal distributions (r_ψ and σ_ψ^2) used in the Metropolis algorithm for hyper-parameter updating are adjusted to get the acceptance rate (R_ψ) between 25–45%. If $R_\psi < 0.25$, $r_\psi = r_\psi / a$, while if $R_\psi > 0.25$, $r_\psi = ar_\psi$, where a is an adjustment coefficient close to but larger than 1. After some experimentation, $a = \sqrt{3-u}$ was chosen as a default approach to get a for a single adjustment cycle, where u is drawn from the (0, 1) uniform distribution. In this way, the value of a vary randomly between $\sqrt{2}$ and 2 every adjustment cycle. σ_ψ^2 is adjusted analogously.

References

Casella, G. & George, E.I. 1992. Explaining the Gibbs sampler. Am. Stat. 46: 167–174. https://doi.org/10.2307/2685208

Green, P. J. (1995). Reversible jump MCMC computation and Bayesian model determination. *Biometrika* 82: 711–732. https://doi.org/10.1093/biome t/82.4.711

Metropolis, N., Rosenbluth, A. W., Rosenbluth, M. N., Teller, A. H., Teller, E. 1953. Equation of State Calculations by Fast Computing Machines. *J. Chem. Phys.* 21: 1087–1092. https://doi.org/10.1063/1.1699114

Pritchard, J. K., Stephens, M., Donnelly, P. 2000. Inference of Population Structure Using Multilocus Genotype Data. *Genetics* 155: 945–959. https://doi.org/10.1093/genetics/155.2.945

Vogl, C., Karhu, A., Moran, G. and Savolainen, O. (2002), High resolution analysis of mating systems: inbreeding in natural populations of *Pinus radiata*. J. *Evol. Biol*. 15: 433-439. https://doi.org/10.1046/j.1420-9101.2002.00404.x

Chybicki, I. J., Oleksa, A., Burczyk, J. 2011. Increased inbreeding and strong kinship structure in Taxus baccata estimated from both AFLP and SSR data. *Heredity* 107: 589–600. https://doi.org/10.1038/hdy.2011.51

Foll, M. & Gaggiotti, O. 2006. Identifying the environmental factors that determine the genetic structure of populations. *Genetics* 174: 875-891. https://doi.org/10.1534/genetics.106.059451

Supplement C. Monte Carlo simulations.

For each independent simulation replicate, we considered N individuals randomly sampled from K discrete populations. First, we independently randomly draw the pre-migration divergence rate (F_{ST}^{j}) of each population from a negative exponential distribution with mean $\mu_{F_{ST}}$. We considered L marker loci, randomly drawing the pre-migration allele frequencies of population j at locus l from a Dirichlet distribution, $E[\mathbf{p}_{jl}]\sim\mathrm{Dir}(\boldsymbol{\theta}_{jl})$, where $\boldsymbol{\theta}_{jl} = \mathbf{q}_{jl}(1 - F_{ST}^{j})/F_{ST}^{j}$. The vector $\mathbf{q}_{jl}$ of global (ancestral) allele frequencies was assumed to follow a discrete uniform distribution. Next, random vectors of expected seed and pollen migration rates for the i-th population, $E[\boldsymbol{\alpha}_i]$ and $E[\boldsymbol{\beta}_i]$, were drawn from Dirichlet distributions specified by, respectively, $(\gamma_\alpha, \lambda_\alpha, \tau_\alpha, b_\alpha)$ and $(\gamma_\beta, \lambda_\beta, \tau_\beta, b_\beta)$ parameter sets, which determine the dispersion (in the case of γ_α and γ_β, see Equation 5 in the main text) and the Dirichlet's mean vector (in the case of the rest of the parameters, see Equations 6 and 7). Finally, every individual was assigned an expected inbreeding coefficient $E[F_i]$, which was randomly drawn from a beta distribution with grand mean μ_F and dispersion γ_F. The described set of parameter expectations constituted the kernel of the data generation process.

We then assumed post-migration sampling with equal effort across populations. For each sampled individual i, we first randomly assigned a sampling location (population) s_i out of the K possible ones. We then randomly draw the population origin of its female and male gametes, $o_i = \{j, k\}$, from the discrete distributions defined by, respectively, $E[\boldsymbol{\alpha}]$ and $E[\boldsymbol{\beta}]$, specifically $j \sim E[\boldsymbol{\alpha}_{s_i}]$ and $k \sim E[\boldsymbol{\beta}_j]$. We thus allowed double migration events, i.e. it was possible that individual i sampled in population s_i originated from a seed that migrated from population j to s_i, and that this seed was in turn sired by migrating pollen from population k to j. After drawing o_i, we adjusted the expected inbreeding of individual i as $E[F_i] = E[F_i] \times \delta_{jk}$, where δ_{jk} is the Kronecker delta ($\delta_{jk} = 1$ if $j = k$, and 0 otherwise), in order to reflect the assumption that alleles can only be identical by descent if they have originated in the same population.

Individual genotypes were then generated using the simulated expected allele frequencies and inbreeding coefficients. In particular, for the l-th locus of the i-th individual, alleles g_{il1} and g_{il2} were randomly drawn sequentially, first the maternal g_{il1} from $E[\mathbf{p}_{jl}]$ and then the paternal g_{il2}, the latter set equal to g_{il1} with probability $E[F_i]$ or drawn from $E[\mathbf{p}_{kl}]$ with probability $1 - E[F_i]$.

The range of parameter values considered and the number of independent replicates for each parameter combination (scenario) are specified in the Methods section of the main text. For each replicate, we estimated the posterior distribution of parameters using the MCMC algorithm described in Supplement B, with 10,000 burn-in cycles followed by 20,000 cycles thinned to every 10th, yielding a final sample of 2,000.

For each parameter and scenario, the expected estimation bias and root mean square error (RMSE) were calculated by comparing the assumed parameter value against the set of posterior median estimates across the simulated data replicates. In scenarios where the distance effects b_α and b_β were estimated, we also tallied the number of independent replicates in which the reversible-jump

MCMC algorithm (see Supplement B) selected the model including b_α (or b_β) as the best model, and the number of replicates it selected the model without it. For any given replicate, we considered as the selected best model the one of the two (i.e., including or excluding b_α or b_β) that was visited more frequently during the sampled MCMC cycles.

Supplement D. Calculation of gene migration rates from seed and pollen migration rates.

Given the vector of seed migration rates $\boldsymbol{\alpha} = \{\alpha_{ij}\}$, where α_{ij} is defined as the probability that individuals in population i originate from seeds dispersed from population j during the previous generation, and given the vector of pollen migration rates $\boldsymbol{\beta} = \{\beta_{ij}\}$, where β_{ij} is the probability that seeds produced in population i are the result of pollination by pollen dispersed from population j during the previous generation, it is then possible to calculate the vector of gene migration rates $\mathbf{m} = \{m_{ij}\}$ by computing

$$m_{ij} = \alpha_{ij}\beta_{jj} + \frac{\alpha_{ij}(1-\beta_{jj})}{2} + \frac{1}{2}\sum_{k\neq j}\alpha_{ik}\beta_{kj} = \frac{1}{2}\left(\alpha_{ij}(1+\beta_{jj}) + \sum_{k\neq j}\alpha_{ik}\beta_{kj}\right)$$

where m_{ij} is the probability that a gene in population i came from population j during the previous generation.

The above definition of recent migration in terms of genes matches the one used in BIMr model (Faubet and Gaggiotti 2008). By contrast, the BAYESASS model of Wilson and Rannala (2003) defines recent migration rates based on the fraction of migrant individuals (m^*) and, assuming small and temporally constant m^*, their prior predicts an expected proportion of first-generation migrant individuals of m^* and a proportion of individuals with one migrant parent of $2m^*$, which translates into an expected proportion of total gene migration that also equals $2m^*$.

Note that in order to be able to consider migration from two different populations (which BAYESASS does not), neither our model nor BIMr distinguish between first- and second-generation migrants.

Second-generation migrants having one parent of single migrant ancestry (e.g. a parent originating from a migrant seed sired by pollen from the same population from where it dispersed) would be genetically indistinguishable from first-generation pollen (gametic) migrants.

References

Faubet P, Gaggiotti OE (2008) A new Bayesian method to identify the environmental factors that influence recent migration. Genetics 178:1491–1504. https://doi.org/10.1534/genetics.107.082560

Wilson GA, Rannala B (2003) Bayesian inference of recent migration rates using multilocus genotypes. Genetics 163:1177–1191. https://doi.org/https://doi.org/10.1093/genetics/163.3.1177

Table S1. Effect of mean population genetic differentiation ($\mu_{F_{ST}}$) and total sample size (N) on the bias and root mean square error (RMSE) of estimates of migration rates, population divergence and inbreeding, assuming microsatellite-type markers, moderate inbreeding ($\mu_F = 0.1$) and no distance effect on migration rates.

		Seed migration (α_{ij})		Pollen migration (β_{ij})		Population divergence (F_{ST}^{j})		Individual inbreeding (F_i)	
$\mu_{F_{ST}}$	N	Bias	RMSE	Bias	RMSE	Bias	RMSE	Bias	RMSE
0.200	1000	−0.0006	0.0144	−0.0003	0.0161	−0.0075	0.0248	−0.0005	0.0673
	500	−0.0006	0.0220	0.0004	0.0218	−0.0101	0.0288	−0.0006	0.0661
	250	−0.0007	0.0307	0.0002	0.0287	−0.0038	0.0271	−0.0052	0.0702
0.100	1000	0.0002	0.0170	−0.0019	0.0222	−0.0044	0.0138	−0.0107	0.0676
	500	0.0000	0.0211	−0.0040	0.0289	−0.0055	0.0181	−0.0102	0.0696
	250	0.0000	0.0311	−0.0044	0.0366	−0.0051	0.0193	−0.0136	0.0697
0.050	1000	0.0019	0.0198	−0.0131	0.0406	−0.0051	0.0102	−0.0185	0.0712
	500	0.0011	0.0297	−0.0140	0.0433	−0.0051	0.0140	−0.0141	0.0699
	250	0.0007	0.0414	−0.0219	0.0582	−0.0100	0.0161	−0.0136	0.0719
0.025	1000	0.0024	0.0303	−0.0267	0.0601	−0.0059	0.0086	−0.0077	0.0688
	500	0.0016	0.0420	−0.0256	0.0589	−0.0052	0.0094	−0.0105	0.0691
	250	0.0034	0.0465	−0.0223	0.0557	−0.0061	0.0123	−0.0121	0.0719

Based on 10 independent replicates per scenario, assuming $L = 20$ loci, 6 alleles/locus, $K = 10$ populations, isolation parameters $\tau_\alpha = \tau_\beta = 0.25$, dispersion parameters $\gamma_\alpha = \gamma_\beta = \gamma_F = 0.1$ and $\gamma_{F_{ST}} = 0.01$.

Table S2. Effect of mean population genetic differentiation ($\mu_{F_{ST}}$) and total sample size (N) on the bias and root mean square error (RMSE) of estimates of migration rates, population divergence and inbreeding, assuming microsatellite-type markers, strong inbreeding (μ_F = 0.2) and no distance effect on migration rates.

		Seed migration (α_{ij})		Pollen migration (β_{ij})		Population divergence (F_{ST}^j)		Individual inbreeding (F_i)	
$\mu_{F_{ST}}$	N	Bias	RMSE	Bias	RMSE	Bias	RMSE	Bias	RMSE
0.200	1000	0.0007	0.0162	0.0001	0.0169	0.0092	0.0247	−0.0025	0.0822
	500	−0.0008	0.0219	0.0003	0.0227	−0.0127	0.0254	−0.0016	0.0843
	250	0.0003	0.0366	−0.0010	0.0370	−0.0095	0.0296	−0.0050	0.0829
0.100	1000	0.0003	0.0165	−0.0031	0.0209	−0.0079	0.0155	−0.0070	0.0849
	500	0.0005	0.0242	−0.0059	0.0330	−0.0069	0.0165	−0.0083	0.0863
	250	−0.0001	0.0324	−0.0073	0.0410	−0.0065	0.0197	−0.0095	0.0866
0.050	1000	0.0021	0.0221	−0.0191	0.0433	−0.0069	0.0115	−0.0108	0.0910
	500	0.0023	0.0316	−0.0190	0.0508	−0.0058	0.0125	−0.0118	0.0896
	250	0.0019	0.0410	−0.0253	0.0602	−0.0115	0.0190	−0.0097	0.0947
0.025	1000	0.0008	0.0299	−0.0237	0.0493	−0.0043	0.0079	−0.0048	0.0901
	500	0.0021	0.0419	−0.0275	0.0576	−0.0066	0.0110	−0.0028	0.0892
	250	0.0002	0.0474	−0.0234	0.0548	−0.0051	0.0131	−0.0098	0.0900

Based on 10 independent replicates per scenario, assuming L = 20 loci, 6 alleles/locus, K = 10 populations, isolation parameters τ_α = τ_β = 0.25, dispersion parameters γ_α = γ_β = γ_F = 0.1 and $\gamma_{F_{ST}}$ = 0.01.

Table S3. Effect of mean population genetic differentiation ($\mu_{F_{ST}}$) and total sample size (N) on the bias and root mean square error (RMSE) of estimates of migration rates, population divergence and inbreeding, assuming SNP-type markers, moderate inbreeding (μ_F = 0.1) and no distance effect on migration rates.

		Seed migration (α_{ij})		Pollen migration (β_{ij})		Population divergence (F_{ST}^j)		Individual inbreeding (F_i)	
$\mu_{F_{ST}}$	N	Bias	RMSE	Bias	RMSE	Bias	RMSE	Bias	RMSE
0.200	1000	0.0002	0.0140	0.0002	0.0158	−0.0066	0.0099	−0.0001	0.0257
	500	−0.0006	0.0233	0.0001	0.0217	−0.0071	0.0097	−0.0001	0.0254
	250	0.0005	0.0244	−0.0007	0.0306	−0.0052	0.0096	−0.0004	0.0255
0.100	1000	0.0004	0.0153	0.0014	0.0154	−0.0020	0.0052	0.0002	0.0243
	500	−0.0004	0.0196	−0.0016	0.0213	−0.0012	0.0053	0.0001	0.0247
	250	0.0016	0.0253	0.0000	0.0262	−0.0033	0.0065	0.0002	0.0252
0.050	1000	−0.0002	0.0158	−0.0001	0.0164	−0.0003	0.0023	−0.0003	0.0242
	500	−0.0002	0.0202	−0.0006	0.0219	−0.0009	0.0032	0.0003	0.0241
	250	−0.0001	0.0272	−0.0011	0.0312	−0.0004	0.0036	−0.0008	0.0248
0.025	1000	0.0007	0.0161	−0.0019	0.0189	−0.0004	0.0020	−0.0024	0.0247
	500	0.0001	0.0225	−0.0034	0.0234	−0.0006	0.0020	−0.0028	0.0242
	250	0.0005	0.0281	−0.0057	0.0387	−0.0015	0.0036	−0.0029	0.0241

Based on 10 independent replicates per scenario, assuming L = 1,000 loci, 2 alleles/locus, K = 10 populations, isolation parameters $\tau_\alpha = \tau_\beta$ = 0.25, dispersion parameters $\gamma_\alpha = \gamma_\beta = \gamma_F$ = 0.1 and $\gamma_{F_{ST}}$ = 0.01.

Table S4. Effect of mean population genetic differentiation ($\mu_{F_{ST}}$) and total sample size (N) on the bias and root mean square error (RMSE) of estimates of migration rates, population divergence and inbreeding, assuming SNP-type markers, strong inbreeding (μ_F = 0.2) and no distance effect on migration rates.

		Seed migration (α_{ij})		Pollen migration (β_{ij})		Population divergence (F_{ST}^j)		Individual inbreeding (F_i)	
$\mu_{F_{ST}}$	N	Bias	RMSE	Bias	RMSE	Bias	RMSE	Bias	RMSE
0.200	1000	0.0002	0.0166	0.0004	0.0151	−0.0060	0.0104	0.0006	0.0275
	500	0.0007	0.0200	0.0001	0.0215	−0.0078	0.0123	0.0005	0.0289
	250	−0.0002	0.0267	0.0006	0.0248	−0.0078	0.0107	0.0004	0.0282
0.100	1000	0.0001	0.0157	0.0011	0.0145	−0.0013	0.0049	−0.0003	0.0260
	500	−0.0005	0.0206	0.0009	0.0194	−0.0021	0.0050	−0.0010	0.0264
	250	−0.0009	0.0290	0.0009	0.0293	−0.0033	0.0059	0.0001	0.0254
0.050	1000	0.0000	0.0169	−0.0001	0.0162	−0.0005	0.0026	−0.0001	0.0253
	500	0.0010	0.0221	−0.0005	0.0205	−0.0008	0.0028	−0.0004	0.0258
	250	0.0010	0.0288	−0.0011	0.0325	−0.0013	0.0044	−0.0006	0.0253
0.025	1000	0.0006	0.0158	−0.0020	0.0192	−0.0006	0.0019	−0.0016	0.0259
	500	−0.0003	0.0244	−0.0054	0.0277	−0.0009	0.0026	−0.0022	0.0268
	250	−0.0005	0.0410	−0.0097	0.0398	−0.0023	0.0042	−0.0013	0.0264

Based on 10 independent replicates per scenario, assuming L = 1,000 loci, 2 alleles/locus, K = 10 populations, isolation parameters τ_α = τ_β = 0.25, dispersion parameters γ_α = γ_β = γ_F = 0.1 and $\gamma_{F_{ST}}$ = 0.01.

Table S5. Effect of the number of populations (K), total sample size (N) and marker type on the bias and root mean square error (RMSE) of estimates of migration rates, population divergence and inbreeding, assuming no inbreeding (μ_F = 0), moderate population genetic differentiation ($\mu_{F_{ST}}$ = 0.10) and no distance effect on migration rates.

			Seed migration (α_{ij})		Pollen migration (β_{ij})		Population divergence (F_{ST}^j)		Individual inbreeding (F_i)	
markers	N	K	Bias	RMSE	Bias	RMSE	Bias	RMSE	Bias	RMSE
SSRs	1000	10	–0.0003	0.0156	0.0000	0.0221	–0.0043	0.0140	0.0019	0.0036
		20	–0.0001	0.0163	0.0000	0.0201	–0.0006	0.0165	0.0017	0.0032
	500	10	–0.0004	0.0220	–0.0007	0.0294	–0.0012	0.0149	0.0027	0.0072
		20	0.0000	0.0243	–0.0015	0.0306	–0.0035	0.0214	0.0027	0.0088
	250	10	0.0000	0.0392	−0.0011	0.0430	–0.0046	0.0195	0.0039	0.0086
		20	–0.0008	0.0312	–0.0015	0.0323	–0.0035	0.0292	0.0036	0.0085
SNPs	1000	10	0.0004	0.0151	−0.0006	0.0157	−0.0014	0.0045	0.0010	0.0018
		20	0.0001	0.0146	0.0001	0.0155	–0.0009	0.0044	0.0009	0.0019
	500	10	–0.0001	0.0217	0.0003	0.0195	–0.0017	0.0048	0.0009	0.0022
		20	–0.0002	0.0176	–0.0004	0.0201	–0.0010	0.0053	0.0011	0.0015
	250	10	–0.0013	0.0303	0.0000	0.0274	−0.0017	0.0060	0.0014	0.0028
		20	0.0002	0.0247	0.0003	0.0252	–0.0020	0.0059	0.0019	0.0028

Based on 10 independent replicates per scenario, assuming either L = 20 loci, 6 alleles/locus (for microsatellite-type markers, SSRs) or L = 1,000 loci, 2 alleles/locus (for SNP-type markers), isolation parameters τ_α = τ_β = 0.25, dispersion parameters γ_α = γ_β = γ_F = 0.1 and $\gamma_{F_{ST}}$ = 0.01.

Table S6. Effect of mean population genetic differentiation ($\mu_{F_{ST}}$) and magnitude of inter-population distance effects on seed and pollen migration (b_α and b_β, respectively) on the bias and root mean square error (RMSE) of estimates of the distance effects and of seed and pollen migration rates (α_{ij} and β_{ij}, respectively), assuming a total sample size of N = 500 individuals. The model ability to identify correctly the presence or absence of distance effects was characterized by the number of independent simulation replicates (*n pos*) in which the reversible-jump MCMC algorithm selected the model including b_α (or b_β) as the best model, which was based on the proportion of times (Pr) it was visited during the sampled MCMC cycles.

			Seed migration						Pollen migration					
$\mu_{F_{ST}}$	b_α	b_β	Bias(α_{ij})	RMSE(α_{ij})	Bias(b_α)	RMSE(b_α)	Pr(b_α)	*n pos*	Bias(β_{ij})	RMSE(β_{ij})	Bias(b_β)	RMSE(b_β)	Pr(b_β)	*n pos*
0.200	2.1274	2.1274	0.0002	0.0218	−0.0484	0.2786	0.9915	10	0.0001	0.0222	−0.1777	0.3816	0.9949	10
		1.2062	−0.0011	0.0218	−0.1272	0.3282	0.9975	10	0.0009	0.0213	0.0272	0.2096	0.9915	10
		0	−0.0006	0.0229	−0.3082	0.4197	0.9977	10	0.0003	0.0227	0.0000	0.0000	0.0574	0
	1.2062	2.1274	0.0001	0.0195	0.2573	0.3703	0.9987	10	−0.0007	0.0212	−0.0713	0.3336	0.9916	10
		1.2062	0.0009	0.0225	0.0328	0.4845	0.9386	9	0.0005	0.0238	0.2774	0.4116	0.9805	10
		0	0.0019	0.0223	0.0323	0.2903	0.9628	10	0.0011	0.0238	−0.0777	0.2457	0.1941	1
	0	2.1274	0.0005	0.0212	0.0000	0.0000	0.0697	0	0.0012	0.0233	−0.0074	0.2205	0.9954	10
		1.2062	0.0004	0.0209	0.0000	0.0000	0.0622	0	0.0003	0.0217	−0.0060	0.6752	0.8273	8
		0	0.0002	0.0227	0.0000	0.0000	0.0618	0	0.0007	0.0215	0.0419	0.4321	0.2263	3
0.100	2.1274	2.1274	0.0011	0.0250	−0.2275	0.4357	0.9935	10	−0.0013	0.0271	−0.1137	0.4201	0.9673	10
		1.2062	−0.0003	0.0229	−0.1644	0.3181	0.9936	10	−0.0010	0.0268	−0.0741	0.4652	0.8149	9
		0	0.0011	0.0245	0.1801	0.4725	0.9902	10	−0.0015	0.0279	0.0000	0.0000	0.0860	0
	1.2062	2.1274	0.0004	0.0217	0.0355	0.2577	0.9545	10	−0.0014	0.0281	−0.0897	0.3823	0.9801	10
		1.2062	0.0000	0.0251	−0.0533	0.5169	0.8907	9	−0.0009	0.0323	0.0039	0.6316	0.8019	8
		0	−0.0010	0.0221	−0.3123	0.4571	0.8184	9	−0.0015	0.0328	0.0000	0.0000	0.0907	0
	0	2.1274	−0.0011	0.0214	0.0000	0.0000	0.0814	0	0.0007	0.0276	−0.0188	0.4361	0.9838	10
		1.2062	0.0001	0.0214	0.0000	0.0000	0.0984	0	−0.0009	0.0270	−0.0105	0.5300	0.8558	9
		0	−0.0003	0.0223	0.0000	0.0000	0.0463	0	−0.0014	0.0301	−0.1074	0.3396	0.1225	1

Table S6 (continued).

$\mu_{F_{ST}}$	b_α	b_β	Seed migration						Pollen migration					
			Bias(α_{ij})	RMSE(α_{ij})	Bias(b_α)	RMSE(b_α)	Pr(b_α)	*n pos*	Bias(β_{ij})	RMSE(β_{ij})	Bias(b_β)	RMSE(b_β)	Pr(b_β)	*n pos*
0.050	2.1274	2.1274	−0.0001	0.0262	−0.1788	0.3725	0.9845	10	−0.0024	0.0448	−0.6654	1.0249	0.7645	8
		1.2062	−0.0008	0.0279	−0.0445	0.5235	0.9898	10	−0.0054	0.0485	−0.6482	1.0911	0.3901	3
		0	0.0004	0.0297	−0.0288	0.3909	0.9512	10	−0.0082	0.0478	0.0000	0.0000	0.1223	0
	1.2062	2.1274	−0.0007	0.0269	−0.1013	0.4504	0.8098	9	−0.0052	0.0496	−1.2001	1.5353	0.6134	5
		1.2062	0.0014	0.0288	0.2864	0.5235	0.9469	10	−0.0071	0.0481	−0.4433	1.0611	0.4518	4
		0	−0.0013	0.0277	0.1973	0.2944	0.9932	10	−0.0044	0.0439	0.0000	0.0000	0.0981	0
	0	2.1274	0.0014	0.0288	0.0000	0.0000	0.0819	0	−0.0021	0.0419	−0.9257	1.3570	0.6211	6
		1.2062	−0.0005	0.0295	−0.1189	0.3758	0.1147	1	−0.0020	0.0499	−0.4223	0.9017	0.4598	5
		0	−0.0025	0.0476	−0.0951	0.3008	0.1189	1	−0.0064	0.0574	0.0000	0.0000	0.1166	0
0.025	2.1274	2.1274	−0.0003	0.0432	−0.0920	0.4103	0.9415	10	−0.0100	0.0604	−1.9176	2.0183	0.2472	1
		1.2062	−0.0020	0.0386	−0.1003	0.2864	0.9296	10	−0.0076	0.0602	−1.0271	1.1591	0.2850	1
		0	−0.0014	0.0389	−0.5715	1.0453	0.8314	8	−0.0037	0.0523	0.0000	0.0000	0.1231	0
	1.2062	2.1274	−0.0016	0.0368	−0.2007	0.7226	0.6233	7	−0.0033	0.0589	−1.4815	1.7837	0.3495	3
		1.2062	−0.0027	0.0440	0.0679	0.6929	0.7125	8	−0.0058	0.0537	−1.0142	1.1663	0.3094	1
		0	−0.0014	0.0413	−0.3908	0.9172	0.5222	5	−0.0068	0.0565	0.0000	0.0000	0.0996	0
	0	2.1274	−0.0012	0.0422	0.0000	0.0000	0.0607	0	−0.0069	0.0582	−1.6611	1.9089	0.3820	2
		1.2062	−0.0016	0.0426	0.5193	0.9869	0.3155	3	−0.0056	0.0576	−0.8508	1.1087	0.2451	2
		0	−0.0039	0.0445	0.0000	0.0000	0.1343	0	−0.0039	0.0519	0.0000	0.0000	0.1310	0

Based on 10 independent replicates per scenario, assuming $L = 20$ loci with 6 alleles/locus, $K = 9$ populations, no inbreeding ($\mu_F = 0$), isolation parameters $\tau_\alpha = \tau_\beta = 0.25$ and dispersion parameters $\gamma_\alpha = \gamma_\beta = \gamma_F = 0.1$.

Table S7. Effect of mean population genetic differentiation ($\mu_{F_{ST}}$) and magnitude of inter-population distance effects on seed and pollen migration (b_α and b_β, respectively) on the bias and root mean square error (RMSE) of estimates of the distance effects and of seed and pollen migration rates (α_{ij} and β_{ij}, respectively), assuming a total sample size of N = 250 individuals. The model ability to identify correctly the presence or absence of distance effects was characterized by the number of independent simulation replicates (*n pos*) in which the reversible-jump MCMC algorithm selected the model including b_α (or b_β) as the best model, which was based on the proportion of times (Pr) it was visited during the sampled MCMC cycles.

			Seed migration						Pollen migration					
$\mu_{F_{ST}}$	b_α	b_β	Bias(α_{ij})	RMSE(α_{ij})	Bias(b_α)	RMSE(b_α)	Pr(b_α)	*n pos*	Bias(β_{ij})	RMSE(β_{ij})	Bias(b_β)	RMSE(b_β)	Pr(b_β)	*n pos*
0.200	2.1274	2.1274	−0.0010	0.0301	−0.1901	0.3234	0.9932	10	−0.0006	0.0310	−0.1347	0.4358	0.9687	10
		1.2062	−0.0011	0.0281	−0.1065	0.2512	0.9931	10	0.0001	0.0333	0.1336	0.7521	0.8383	8
		0	0.0009	0.0291	−0.2524	0.3239	0.9955	10	−0.0026	0.0355	0.0460	0.5054	0.2601	2
	1.2062	2.1274	0.0002	0.0309	−0.0635	0.6031	0.8819	8	−0.0012	0.0268	−0.2002	0.3506	0.9862	10
		1.2062	−0.0001	0.0282	−0.1153	0.4443	0.8180	9	−0.0034	0.0305	−0.1422	0.4593	0.8236	9
		0	−0.0011	0.0295	0.0366	0.2938	0.8830	10	0.0003	0.0310	0.0000	0.0000	0.0948	0
	0	2.1274	0.0018	0.0257	−0.1200	0.3796	0.1274	1	−0.0002	0.0267	−0.2384	0.4180	0.9921	10
		1.2062	0.0006	0.0263	0.0000	0.0000	0.1231	0	−0.0002	0.0288	−0.2357	0.7659	0.6762	7
		0	−0.0004	0.0325	0.0000	0.0000	0.0832	0	−0.0006	0.0295	0.0000	0.0000	0.0540	0
0.100	2.1274	2.1274	−0.0007	0.0319	0.0718	0.3651	0.9714	10	−0.0009	0.0400	−0.1796	0.7934	0.9042	9
		1.2062	0.0010	0.0432	−0.2598	0.4042	0.9902	10	−0.0033	0.0467	−0.5130	0.8693	0.5581	5
		0	−0.0001	0.0304	−0.0127	0.3672	0.9843	10	0.0000	0.0402	0.0000	0.0000	0.0646	0
	1.2062	2.1274	0.0009	0.0298	0.1544	0.3237	0.9645	10	−0.0002	0.0383	−0.2001	0.5172	0.9532	10
		1.2062	−0.0009	0.0310	−0.1026	0.4655	0.8640	9	−0.0011	0.0377	−0.2134	0.9786	0.5847	6
		0	0.0001	0.0332	−0.0656	0.4774	0.7889	9	0.0000	0.0380	0.0000	0.0000	0.1110	0
	0	2.1274	0.0000	0.0319	0.0000	0.0000	0.1061	0	0.0002	0.0363	−0.2448	0.4133	0.9106	10
		1.2062	0.0012	0.0357	−0.1258	0.3977	0.2036	1	−0.0047	0.0472	−0.3204	0.8389	0.5823	6
		0	0.0018	0.0347	0.0000	0.0000	0.0417	0	−0.0041	0.0450	−0.0318	0.6262	0.2395	2

Table S7 (continued).

$\mu_{F_{ST}}$	b_α	b_β	Seed migration Bias(α_{ij})	RMSE(α_{ij})	Bias(b_α)	RMSE(b_α)	Pr(b_α)	*n pos*	Pollen migration Bias(β_{ij})	RMSE(β_{ij})	Bias(b_β)	RMSE(b_β)	Pr(b_β)	*n pos*
0.050	2.1274	2.1274	0.0006	0.0370	−0.3552	0.8340	0.8233	9	−0.0050	0.0519	−1.3720	1.6608	0.4395	4
		1.2062	0.0015	0.0472	−0.2988	0.3879	0.9737	10	−0.0054	0.0453	−0.6424	1.0755	0.3047	3
		0	0.0016	0.0393	−0.4177	1.0156	0.8636	8	−0.0053	0.0532	0.0000	0.0000	0.1554	0
	1.2062	2.1274	0.0011	0.0482	−0.0188	0.6726	0.7353	8	−0.0041	0.0535	−1.7120	1.9039	0.3379	2
		1.2062	−0.0016	0.0400	−0.4827	1.0207	0.5886	4	−0.0048	0.0561	−0.8422	1.1132	0.3181	2
		0	0.0012	0.0385	−0.7943	1.0287	0.4324	3	−0.0043	0.0588	0.0000	0.0000	0.1488	0
	0	2.1274	−0.0006	0.0370	0.0000	0.0000	0.0975	0	−0.0058	0.0577	−1.0731	1.5056	0.4509	5
		1.2062	−0.0031	0.0446	0.0000	0.0000	0.1981	0	−0.0043	0.0649	−0.7571	1.1787	0.2895	2
		0	−0.0041	0.0496	0.0000	0.0000	0.1309	0	−0.0051	0.0556	0.0000	0.0000	0.1239	0
0.025	2.1274	2.1274	−0.0045	0.0511	−0.5586	1.0176	0.7678	8	−0.0063	0.0598	−2.1274	2.1274	0.1740	0
		1.2062	−0.0025	0.0508	−0.9513	1.3870	0.5683	6	−0.0083	0.0556	−1.2062	1.2062	0.1455	0
		0	−0.0022	0.0457	−1.0512	1.5065	0.5081	5	−0.0062	0.0480	0.0000	0.0000	0.1175	0
	1.2062	2.1274	0.0001	0.0448	0.0940	0.6766	0.6875	8	−0.0051	0.0632	−1.9521	2.0217	0.1599	1
		1.2062	−0.0042	0.0478	−0.6622	1.0719	0.3823	3	−0.0044	0.0652	−1.2062	1.2062	0.1196	0
		0	−0.0055	0.0509	−0.6143	0.9632	0.3912	4	−0.0060	0.0586	0.0000	0.0000	0.1459	0
	0	2.1274	−0.0051	0.0448	0.0000	0.0000	0.1712	0	−0.0035	0.0590	−2.1274	2.1274	0.1251	0
		1.2062	−0.0037	0.0510	0.0000	0.0000	0.1283	0	−0.0042	0.0562	−1.2062	1.2062	0.1238	0
		0	−0.0046	0.0525	0.0000	0.0000	0.1697	0	−0.0059	0.0582	0.0000	0.0000	0.1044	0

Based on 10 independent replicates per scenario, assuming L = 20 loci with 6 alleles/locus, K = 9 populations, no inbreeding (μ_F = 0), isolation parameters $\tau_\alpha = \tau_\beta$ = 0.25 and dispersion parameters $\gamma_\alpha = \gamma_\beta = \gamma_F$ = 0.1.

Table S8. Effect of the number of populations (K) and total sample size (N) on the bias and root mean square error (RMSE) of estimates of distance effects on seed and pollen migration (b_α and b_β, respectively) and of seed and pollen migration rates (α_{ij} and β_{ij}, respectively), assuming moderate population genetic differentiation ($\mu_{F_{ST}}$ = 0.10) and weak isolation by distance ($b_\alpha = b_\beta$ = 1.2062). The model ability to identify correctly the presence or absence of distance effects was characterized by the number of independent simulation replicates (*n pos*) in which the reversible-jump MCMC algorithm selected the model including b_α (or b_β) as the best model, which was based on the proportion of times (Pr) it was visited during the sampled MCMC cycles.

		Seed migration						Pollen migration					
N	K	Bias(α_{ij})	RMSE(α_{ij})	Bias(b_α)	RMSE(b_α)	Pr(b_α)	*n pos*	Bias(β_{ij})	RMSE(β_{ij})	Bias(b_β)	RMSE(b_β)	Pr(b_β)	*n pos*
1000	9	−0.0003	0.0170	0.0882	0.3737	0.9752	10	0.0006	0.0218	0.0514	0.5221	0.8999	9
	18	0.0004	0.0157	−0.0327	0.1439	0.9997	10	−0.0004	0.0212	−0.0173	0.2133	0.9260	10
500	9	0.0000	0.0251	−0.0533	0.5169	0.8907	9	−0.0009	0.0323	0.0039	0.6316	0.8019	8
	18	−0.0003	0.0208	−0.0008	0.4544	0.8789	9	−0.0002	0.0259	−0.2662	0.5741	0.6786	8
250	9	−0.0009	0.0310	−0.1026	0.4655	0.8640	9	−0.0011	0.0377	−0.2134	0.9786	0.5847	6
	18	0.0003	0.0270	0.0264	0.4194	0.8959	9	−0.0009	0.0332	−0.7113	0.9403	0.4244	4

Based on 10 independent replicates per scenario, assuming L = 20 loci with 6 alleles/locus, no inbreeding (μ_F = 0), isolation parameters $\tau_\alpha = \tau_\beta$ = 0.25 and dispersion parameters $\gamma_\alpha = \gamma_\beta = \gamma_F$ = 0.1.

Table S9. Posterior estimates of recent seed (α_{jk}) and pollen (β_{jk}) migration rates among nine *Taxus baccata* remnant populations on the Low Beskids (Poland). The posterior distribution of each parameter was used to calculate the Median, standard error (SE), and lower and upper limits of the 95% highest posterior density intervals (HPDI_L and HPDI_U, respectively).

Recipent population (*j*)	Source population (*k*)	Distance [km]	α_{jk}				β_{jk}			
			Median	SE	HPDI_L	HPDI_U	Median	SE	HPDI_L	HPDI_U
Ig	Le	7.385	0.0002	0.0012	0	0.0004	0.0025	0.0058	0	0.0135
Ig	Lm	8.228	0.0001	0.0010	0	0.0003	0.0021	0.0051	0	0.0122
Ig	Lw	9.675	0.0006	0.0033	0	0.0019	0.0336	0.0239	0	0.0774
Ig	Nw	4.570	0.0001	0.0010	0	0.0002	0.0038	0.0061	0	0.0174
Ig	Pa	10.324	0.0005	0.0027	0	0.0022	0.0100	0.0167	0	0.0465
Ig	Sr	11.240	0.0003	0.0026	0	0.0005	0.0069	0.0133	0	0.0344
Ig	Wa	2.614	0.0001	0.0009	0	0.0002	0.0056	0.0074	0	0.0200
Ig	Za	16.550	0.0001	0.0011	0	0.0002	0.0006	0.0026	0	0.0030
Le	Ig	7.385	0.0001	0.0010	0	0.0002	0.0010	0.0034	0	0.0056
Le	Lm	0.848	0.0005	0.0030	0	0.0011	0.0680	0.0378	0	0.1332
Le	Lw	2.338	0.0005	0.0027	0	0.0013	0.0061	0.0108	0	0.0272
Le	Nw	9.619	0.0002	0.0011	0	0.0003	0.0004	0.0021	0	0.0018
Le	Pa	3.568	0.0001	0.0010	0	0.0002	0.0030	0.0076	0	0.0153
Le	Sr	4.147	0.0002	0.0014	0	0.0004	0.0048	0.0091	0	0.0237
Le	Wa	8.947	0.0001	0.0012	0	0.0001	0.0006	0.0025	0	0.0033
Le	Za	10.115	0.0001	0.0013	0	0.0002	0.0004	0.0019	0	0.0018
Lm	Ig	8.228	0.0003	0.0023	0	0.0006	0.0009	0.0038	0	0.0044
Lm	Le	0.848	0.0011	0.0065	0	0.0038	0.0483	0.0398	0	0.1221
Lm	Lw	1.498	0.0004	0.0028	0	0.0009	0.0118	0.0188	0	0.0485
Lm	Nw	10.418	0.0004	0.0030	0	0.0003	0.0008	0.0034	0	0.0039
Lm	Pa	2.913	0.0004	0.0026	0	0.0008	0.0063	0.0137	0	0.0318
Lm	Sr	3.363	0.0003	0.0024	0	0.0005	0.0032	0.0081	0	0.0174
Lm	Wa	9.740	0.0004	0.0034	0	0.0005	0.0007	0.0035	0	0.0036
Lm	Za	9.402	0.0004	0.0028	0	0.0002	0.0011	0.0052	0	0.0060
Lw	Ig	9.675	0.0003	0.0020	0	0.0006	0.0009	0.0034	0	0.0055
Lw	Le	2.338	0.0002	0.0017	0	0.0004	0.0275	0.0311	0	0.0922
Lw	Lm	1.498	0.0004	0.0030	0	0.0007	0.0333	0.0382	0	0.1140
Lw	Nw	11.902	0.0002	0.0011	0	0.0003	0.0004	0.0018	0	0.0019
Lw	Pa	1.877	0.0003	0.0017	0	0.0005	0.0184	0.0168	0	0.0520
Lw	Sr	1.954	0.0002	0.0015	0	0.0003	0.0193	0.0220	0	0.0628
Lw	Wa	11.063	0.0002	0.0013	0	0.0004	0.0025	0.0062	0	0.0158

Table S9 (continued).

Recipent population (j)	Source population (k)	Distance [km]	α_{jk}				β_{jk}			
			Median	SE	HPDI_L	HPDI_U	Median	SE	HPDI_L	HPDI_U
Lw	Za	8.058	0.0002	0.0013	0	0.0003	0.0081	0.0121	0	0.0336
Nw	Ig	4.570	0.0003	0.0026	0	0.0006	0.0083	0.0120	0	0.0317
Nw	Le	9.619	0.0003	0.0028	0	0.0005	0.0027	0.0064	0	0.0145
Nw	Lm	10.418	0.0003	0.0019	0	0.0004	0.0026	0.0067	0	0.0123
Nw	Lw	11.902	0.0004	0.0024	0	0.0005	0.0023	0.0062	0	0.0126
Nw	Pa	13.106	0.0004	0.0024	0	0.0007	0.0018	0.0050	0	0.0114
Nw	Sr	13.767	0.0003	0.0021	0	0.0006	0.0015	0.0046	0	0.0089
Nw	Wa	6.711	0.0003	0.0027	0	0.0005	0.0046	0.0086	0	0.0218
Nw	Za	19.643	0.0003	0.0021	0	0.0003	0.0011	0.0043	0	0.0064
Pa	Ig	10.324	0.0003	0.0029	0	0.0002	0.0050	0.0150	0	0.0329
Pa	Le	3.568	0.0003	0.0019	0	0.0004	0.0054	0.0112	0	0.0281
Pa	Lm	2.913	0.0003	0.0024	0	0.0007	0.0046	0.0102	0	0.0216
Pa	Lw	1.877	0.0003	0.0025	0	0.0003	0.0107	0.0161	0	0.0421
Pa	Nw	13.106	0.0002	0.0017	0	0.0003	0.0003	0.0017	0	0.0014
Pa	Sr	1.148	0.0046	0.0159	0	0.0324	0.1161	0.0515	0.0223	0.2132
Pa	Wa	11.311	0.0003	0.0019	0	0.0003	0.0005	0.0023	0	0.0031
Pa	Za	6.556	0.0003	0.0028	0	0.0004	0.0012	0.0040	0	0.0074
Sr	Ig	11.240	0.0007	0.0034	0	0.0037	0.0036	0.0094	0	0.0235
Sr	Le	4.147	0.0009	0.0046	0	0.0032	0.0247	0.0306	0	0.0873
Sr	Lm	3.363	0.0003	0.0023	0	0.0004	0.0107	0.0172	0	0.0494
Sr	Lw	1.954	0.0005	0.0030	0	0.0021	0.0319	0.0311	0	0.0886
Sr	Nw	13.767	0.0002	0.0013	0	0.0003	0.0004	0.0020	0	0.0018
Sr	Pa	1.148	0.0791	0.0317	0.0214	0.1383	0.0714	0.0369	0	0.1339
Sr	Wa	12.353	0.0002	0.0012	0	0.0002	0.0004	0.0017	0	0.0020
Sr	Za	6.106	0.0002	0.0011	0	0.0003	0.0012	0.0038	0	0.0069
Wa	Ig	2.614	0.0001	0.0008	0	0.0003	0.0103	0.0089	0	0.0273
Wa	Le	8.947	0.0001	0.0008	0	0.0002	0.0282	0.0179	0	0.0609
Wa	Lm	9.740	0.0001	0.0009	0	0.0003	0.0044	0.0095	0	0.0247
Wa	Lw	11.063	0.0001	0.0008	0	0.0001	0.0026	0.0066	0	0.0150
Wa	Nw	6.711	0.0001	0.0009	0	0.0001	0.0014	0.0030	0	0.0071
Wa	Pa	11.311	0.0001	0.0009	0	0.0002	0.0011	0.0031	0	0.0068
Wa	Sr	12.353	0.0001	0.0006	0	0.0001	0.0008	0.0026	0	0.0046
Wa	Za	17.049	0.0001	0.0008	0	0.0001	0.0004	0.0015	0	0.0027
Za	Ig	16.550	0.0000	0.0003	0	0.0000	0.0020	0.0038	0	0.0105
Za	Le	10.115	0.0001	0.0004	0	0.0001	0.0065	0.0091	0	0.0257
Za	Lm	9.402	0.0001	0.0004	0	0.0001	0.0034	0.0054	0	0.0153
Za	Lw	8.058	0.0001	0.0004	0	0.0001	0.0104	0.0078	0	0.0248
Za	Nw	19.643	0.0000	0.0003	0	0.0000	0.0004	0.0012	0	0.0023
Za	Pa	6.556	0.0000	0.0003	0	0.0001	0.0024	0.0028	0	0.0083
Za	Sr	6.106	0.0001	0.0004	0	0.0001	0.0036	0.0047	0	0.0134
Za	Wa	17.049	0.0000	0.0005	0	0.0000	0.0003	0.0010	0	0.0017

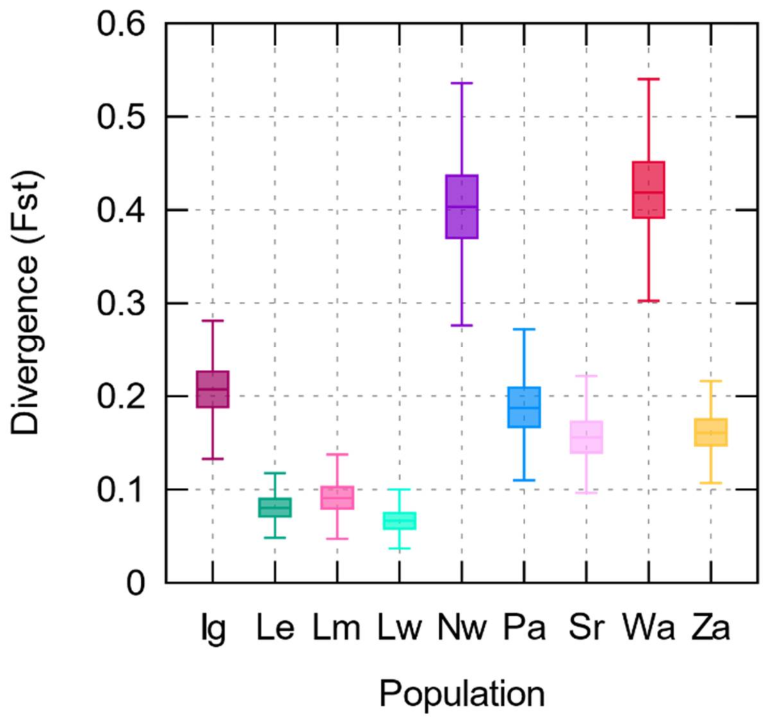

Figure S1. Posterior estimates of pre-migration divergence rates (F_{ST}^{j}) for nine remnant populations of *Taxus baccata*. The box-and-whisker plots show the 90th and 10th percentiles, the median and the first and third quartiles of the posterior distribution for each population.

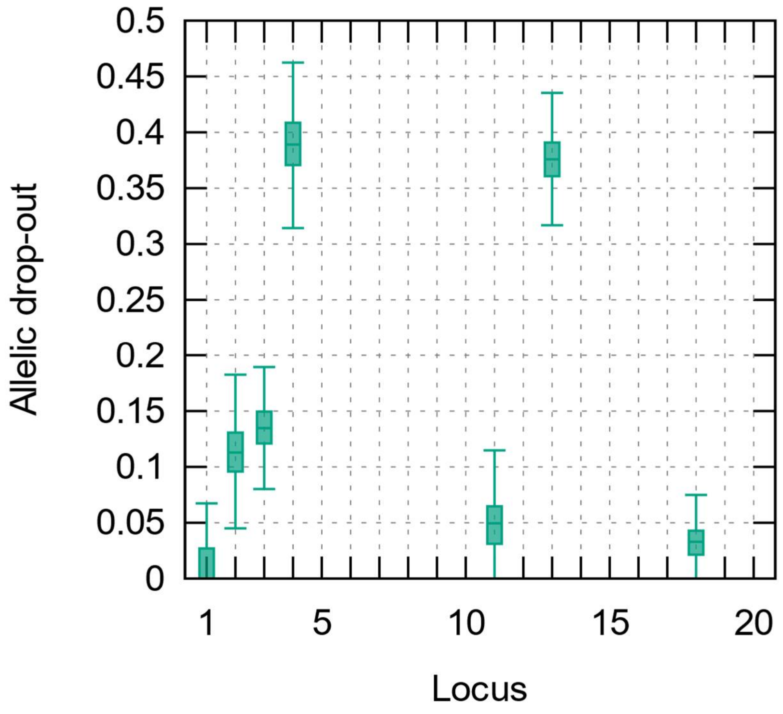

Figure S2. Posterior estimates of allelic drop-out rates for the 20 microsatellite loci used in the *Taxus baccata* case study. The box-and-whisker plots show the 90th and 10th percentiles, the median and the first and third quartiles of the posterior distribution for each locus. The estimated posterior distribution for most loci was very close to zero and is not visible.
